# Combined Mutation Screening of NKX2-5, GATA4, and TBX5 in Congenital Heart Disease: Multiple Heterozygosity and Novel Mutations

**DOI:** 10.1111/j.1747-0803.2011.00573.x

**Published:** 2012-03

**Authors:** Javier T Granados-Riveron, Mark Pope, Frances A Bu'Lock, Christopher Thornborough, Jacqueline Eason, Kerry Setchfield, Ami Ketley, Edwin P Kirk, Diane Fatkin, Michael P Feneley, Richard P Harvey, J David Brook

**Affiliations:** * Institute of Genetics, School of Biology, University of Nottingham, Queen's Medical CentreNottingham, UK; † Department of Paediatric Cardiology, Glenfield HospitalLeicester, UK; ‡ Clinical Genetics Service, City HospitalNottingham, UK; § Developmental and Stem Cell Biology Division, Victor Chang Cardiac Research InstituteDarlinghurst, New South Wales, Australia; ¶ Department of Medical Genetics, Sydney Children's HospitalRandwick, New South Wales, Australia; ** School of Women's and Children's Health, Faculty of Medicine, University of New South WalesNew South Wales, Australia; †† Faculty of Medicine, University of New South WalesNew South Wales, Australia; ‡‡ Cardiology Department, St. Vincent's HospitalDarlinghurst, New South Wales, New South Wales, Australia

**Keywords:** Congenital Heart Disease, Mutations, Multiple Heterozygosity

## Abstract

**Background:**

Variants of several genes encoding transcription modulators, signal transduction, and structural proteins are known to cause Mendelian congenital heart disease (CHD). *NKX2-5* and *GATA4* were the first CHD-causing genes identified by linkage analysis in large affected families. Mutations of *TBX5* cause Holt–Oram syndrome, which includes CHD as a clinical feature. All three genes have a well-established role in cardiac development.

**Design:**

In order to investigate the possible role of multiple mutations in CHD, a combined mutation screening was performed in *NKX2-5*, *GATA4*, and *TBX5* in the same patient cohort. Samples from a cohort of 331 CHD patients were analyzed by polymerase chain reaction, double high-performance liquid chromatography and sequencing in order to identify changes in the *NKX2-5*, *GATA4*, and *TBX5* genes.

**Results:**

Two cases of multiple heterozygosity of putative disease-causing mutations were identified. One patient was found with a novel L122P *NKX2-5* mutation in combination with the private A1443D mutation of *MYH6*. A patient heterozygote for a D425N GATA4 mutation carries also a private mutation of the *MYH6* gene (V700M).

**Conclusions:**

In addition to reporting two novel mutations of *NKX2-5* in CHD, we describe families where multiple individual mutations seem to have an additive effect over the pathogenesis of CHD. Our findings highlight the usefulness of multiple gene mutational analysis of large CHD cohorts.

## Introduction

Congenital heart disease (CHD) is a complex trait, as both environmental and genetic factors have been implicated in its pathogenesis. The etiology of CHD has a strong genetic component, as shown by extensive epidemiological studies in large series of consecutive births.^1^,^2^ In approximately one in four cases, CHD occurs associated with other congenital anomalies within a single-gene disorder (e.g., Holt–Oram syndrome), sporadic malformative complex (e.g., VACTERL association), or as a consequence of a chromosomal abnormality (e.g., trisomy 21).^3^ Cases of isolated CHD appear mainly as sporadic events. However, a small fraction present as familial cases, often showing Mendelian segregation with widely variable penetrance.^4^ The first genes involved in Mendelian isolated CHD, *NKX2-5* and *GATA4*, which encode transcription factors, were identified by genetic linkage studies in large affected families.^5^,^6^

The mammalian *NKX2-5* gene was discovered during a screening for mouse homologues of *tinman*,^7^,^8^ a *Drosophila* gene essential for cardiac development,^9^,^10^ the product of which has been categorized as a Class I NK-2 homeodomain protein.^11^ This group of transcription factors bind to the 5′-CAAGTG-3′ motif in target promoters.^12^ Murine NKX2-5 is expressed from day 7 in the cardiac primordia^8^ and is an early marker of both embryonic heart fields.^13^ Although mutations of NKX2-5 are associated with a wide spectrum of CHDs and thyroid dysgenesis,^14^ most manifest as atrial septal defects (ASDs) and atrioventricular block.^15^

The product of *GATA4* belongs to the zinc-finger family of transcription factors and, as the other GATA proteins, it binds to the 5′-(A/C/T)GATA(A/G)-3′ motif within its target sequences.^16^ The mammalian *GATA4* gene was identified in a screen of zinc-finger encoding cDNAs in a mouse library,^17^ and it was first suspected to be implicated in CHD when a deletion^18^ and a duplication^19^ of the chromosomal segment in which it is contained were discovered in patients with cardiac malformation. Most mutations of *GATA4* occur in the segments of the gene encoding the zinc-finger motifs^16^ and have been related to diverse types of CHDs.^20^

Mutations of *TBX5* cause Holt–Oram syndrome, the most common of the heart–hand syndromes.^21^,^22^ If strict criteria are used in Holt–Oram syndrome diagnosis (i.e., at least one family member with radial ray defect and cardiac septal defects), mutation of *TBX5* is found in more than 70% of cases.^23^ The vertebrate *TBX5* gene was discovered in a screen of mouse cDNA clones using a probe complementary to the TBX2 T-box region.^24^ It encodes a transcription factor and alternative splicing regulator^25^ expressed in the developing heart, among other tissues.^21^ Except for somatic mutations,^26^ no variants of *TBX5* have been found to cause nonsyndromic CHD.

Physical and functional interaction to modulate transcription of cardiac genes has been documented between the NKX2-5, GATA4, and TBX5 proteins.^5^,b27,^28^ In order to evaluate the contribution to multiple mutations in these genes in the pathogenesis of CHD, we conducted a mutational scan of the *NKX2-5*, *GATA4*, and *TBX5* genes in a large cohort of sporadic nonsyndromic CHD cases that complements the findings of our previous analysis of *MYH6* in the same cohort.^29^ We found two novel mutations of the *NKX2-5* gene and two instances in which the distribution of changes in families is consistent with additive effects of variants in different genes in the pathogenesis of CHD.

## Methods

### Patients and Samples

The patient cohort comprised 331 patients with a wide variety of CHDs. Peripheral blood samples from all participants were taken after informed consent and approval of the project by the local ethics committees. Genomic DNA was purified from blood using the QIAmp DNA blood Maxi kit (Qiagen, Hilden, Germany) following the manufacturer directions. Anonymous human control DNA panels were obtained from the European Collection of Cell Culture (Salisbury, UK).

### Denaturing High Performance Liquid Chromatography (dHPLC)

Mutational analysis by dHPLC was performed as described previously.^30^ Briefly, to analyze the combined 18 exons of the *NKX2-5*, *GATA4*, and *TBX5* genes, 35 polymerase chain reaction (PCR) amplicons were designed. Most of the amplicons spanned individual exons and short segments of flanking introns to each side to detect mutations of splicing regulatory elements. Large exons were spanned by two overlapping amplicons. A pair of PCR primers was designed for each amplicon (see Table 1). PCR reactions were performed using patient and control DNA samples following standard protocols. A final hybridization step for heteroduplex formation was carried out heating the PCR products to 95°C and cooling down 1.5°C per minute until a temperature of 25°C was reached. Sequences of individual amplicons were processed by the Navigator software (Transgenomic, Omaha, NE, USA) in order to plot the melting profile of each DNA segment to determine the optimal dHPLC temperatures. PCR products were analyzed on the dHPLC WAVE System (Transgenomic). PCR products displaying a trace indicative of heterozygosity were sequenced by standard protocols. Novel potentially deleterious variants were screened by dHPLC in samples from 384 ethnically matched control subjects.

**Table 1:** Summary of the Primers, Size, Annealing Temperature (Ta) of the Amplicons Used for the PCR Amplification of the *NKX2-5*, *GATA4*, and *TBX5* Genes and Temperatures Used in dHPLC

Amplicon	Forward Primer	Reverse Primer	Size (bp)	Ta	dHPLC Temperatures
NKX2.5-E1P1	tgacacgaaactgctcatcg	gtaggcctctggcttgaagg	416	56.6	63.3, 65.3, 67.3
NKX2.5-E1P2	ctggcgctgtgagactgg	agtttcttggggacgaaagc	422	56	62, 63.6, 66.2, 67.4
NKX2.5-E2P1	caagccgctcttaccaagc	cgttataaccgtagggattgagg	467	59.6	62.4, 65.6, 66.4
NKX2.5-E2P2	ccatgcctaggggactcg	gggggacagctaagacacc	530	60.9	62.2, 63.9
NKX2.5-E2P3	attcactcctgcggagacc	tcaatttgctcagggaatgc	461	54.1	59.3, 62.8, 64, 66.3
GATA4-E1	gtagcacttgggcattttcc	ctacctccagacaagcaaagg	389	58.4	62.1, 64.3, 67.7
GATA4-E2P1	gtgggttctgaaagctctgg	cctcggtgtcctctctctcc	497	58.4	57.4, 59.6, 62.3, 65.1
GATA4-E2P2	cacgcatattatcgttgttgc	gccctggaggtaggacagg	267	54.7	64.3, 66.1, 68.8
GATA4-E2P3	cgtcctcgccagtctacg	gtccccgggaaggagaag	586	60.9	66.2, 68.2, 68.8
GATA4-E3	aaagggcattgtttctgtgc	agaggatgtcccaccaagg	344	54.1	58.6, 61.5, 64.1, 64.7
GATA4-E4	gagttaggtgccgtcacagg	gagagatgggcatcagaagg	336	60.3	63.2, 65.1
GATA4-E5	caggtgtgtgtctttcaatgc	tgattcttaggcactctgagg	229	57.7	58.5, 60.5
GATA4-E6	ccggctgttcgtttgtcc	ctctgggactctgcagtcg	269	59.7	62.6, 65.1
GATA4-E7P1	cagcctagacctccaaagc	acaggagagatgcagtgtgc	499	59.1	60.8, 61.4, 62.2
GATA4-E7P2	gacaatctggttaggggaagc	ccagctgcattttgatgagg	470	58.4	59.4, 61.5, 63.3
GATA4-E7P3	gccctgcatccctaatacc	cagcccttgggacactcc	482	59.8	58.2, 61.2
GATA4-E7P4	agtctggcaagcactcagc	ccagtaggattttggagtgagg	484	59.4	61.2, 62.2
GATA4-E7P5	ctgcacattgctgtttctgc	ctacacggcctcaagattcc	384	58.3	56.5, 58.1, 60.6
TBX5-E1P1	ggtattcatttgcccagagc	cccagtaaaataaagaggcaacc	478	57.9	53.6, 59.2, 63.5, 64.9
TBX5-E1P2	ccagccaaacgtgacagc	gccaagtgcaaagagaaacc	390	57.8	57.8, 60.4, 62.8
TBX5-E2	tttctctcgttctctctctgtcc	cagactctgactttgatctctgc	297	60.2	62.8, 66.2
TBX5-E3	gtgttttgggggagtttgg	gccaccttttcttcttcacc	243	57	58.9, 60.4
TBX5-E4	gaggctgccttaaaatactgg	aactttttgggagaaggttcc	248	56.7	57.7, 60.2
TBX5-E5	ctggtgcgtgaactgaagc	gaggacaagagggagacaagg	282	60.3	62.7, 65.1
TBX5-E6	gggagcagggttttatctgg	tgcaaaagaaagagcagacg	280	54.3	54.6, 57.8, 61.1
TBX5-E7	tggcttaatttgcttcttttgg	ggttgctgctggcttacc	294	53.4	56.5, 58.9
TBX5-E8	tctctcacacctggttcagc	atactcctcaccccctcacc	390	60.3	58.7, 61.3
TBX5-E9P1	ttggccaaataactgtctcc	gctggaacattccctctcc	465	54.1	56.3, 60.5, 63.8
TBX5-E9P2	acttctccgctcacttcacc	tttttaaaattgtggtttcaagc	474	50.3	55.6, 59, 61.3, 63.5
TBX5-E9P3	ggacaagatttttcatttcacc	ggtaggtgcttttcttagtcaagg	496	53.4	52.8, 56.4, 58, 59.3
TBX5-E9P4	ggacccagtcccttatttgg	tttaatcagggagaatatttatttt	481	49.9	56.9, 58, 59.2
TBX5-E9P5	tggcctatagcttcccttcc	ctcttggccagctcctatgc	482	60.3	53.1, 54.5, 56.7, 58.4
TBX5-E9P6	tgtgtaagtaaagtgttatggtagg	aaagagacataatcgcataggg	361	56.9	52.1, 54.2, 57
TBX5-E9P7	aagagaacagggtaagatgtgagg	ttcctgtttcctccaattcc	276	54.2	56.6, 58.7

PCR, polymerase chain reaction; dHPLC, denaturing high performance liquid chromatography.

## Results

**Figure 1:**
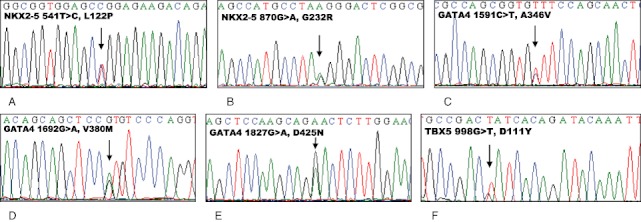
Sequence traces showing the nonsynonymous nucleotide changes causing amino acid replacements in the NKX2.5, GATA4, and TBX5 proteins. The changes are indicated with arrows.

We identified two novel nonsynonymous mutations in *NKX2-5* compared with sequence accession number NM_004387.2 for the cDNA and NP_004378.1 for the protein. In exon 2, a 541T>C transition (L122P) was discovered in a patient with secundum ASD (Figure 1A). The mutation was transmitted by the unaffected father. The same patient also harbors a private mutation we reported previously (a 4395C>A transversion resulting in A1443D) in exon 30 of the *MYH6* gene, which was transmitted by the mother.^29^ A 870G>A (G232R) mutation (Figure 1B) was also identified in exon 2 of a patient with pulmonary valve stenosis that was transmitted by the apparently unaffected mother. The G232R replaces an uncharged glycine residue with a positively charged arginine residue. Neither *NKX2-5* mutation was found in 384 ethnically matched control subjects (768 chromosomes).

Three rare and one common nonsynonymous variants of *GATA4* were found in our CHD cohort. They are identified according to their positions in sequence accession number NM_002052.3 (cDNA) and NP_002043.2 (protein). A 1591C>T transition (A346V) (Figure 1C) was discovered within exon 6 in a patient with transposition of the great arteries. A346V has been previously described in a patient with endocardial cushion defect.^20^ Also within exon 6, a transition 1692G>A (V380M) was identified in a patient with a large ventricular septal defect (VSD) (Figure 1D). V380M has been reported as a pathogenic mutation in CHD in one study,^31^ but another study reports the same variant in 3 out of 318 control subjects.^32^ V380M was not found in our control cohort of 384 ethnically matched subjects. In exon 7, an 1827G>A (D425N) transition was found in a patient with a large patent foramen ovale (Figure 1E). The patient with this D425N mutation has been reported recently,^33^ but no further family data was published. The same mutation has been reported previously in three CHD patients.^34^,^35^ The patient also carries a private mutation in exon 18 of *MYH6*, a 2165G>A transition (V700M).^29^ Two clinically normal sons are both heterozygous for the *GATA4* mutation but do not carry the *MYH6* mutation.

Analysis of the *TBX5* gene revealed one synonymous variant in our patient cohort. In exon 4, a transversion 998G>T (cDNA sequence accession NM_000192.3) (Figure 1F) resulting in D111Y (protein sequence accession NP_000183.2) was discovered in a patient with double outlet right ventricle, large ventricular septal defect, large atrial septal defect, and patent ductus arteriosus. We also found this variant in 3 out of 384 ethnically matched control samples.

## Discussion

We have conducted a mutational scan of the *NKX2-5*, *GATA4*, and *TBX5* genes in a large cohort of patients with a wide variety of CHDs. We have identified two novel changes of NKX2-5 (L122P, G232R), one for TBX5 (D111Y), and three previously known variants of GATA4 (A346V, V380M, and D425N).

**Figure 2:**
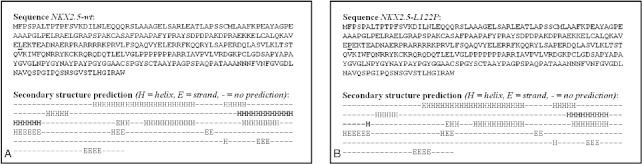
nnpredict output showing the prediction of the structural consequence of the L122P mutation in *NKX2.5*. The panels show the sequence of the wild-type (A) and mutant (B) NKX2.5 proteins. The relevant residues at position 122 are underlined. The secondary structure prediction is shown for each sequence. The fourth helix predicted in the wild-type sequence is 18 residues long (in bold). An eight-residue stretch within that segment is predicted to adopt a different configuration in the mutant peptide (H, helix, E, strand, -. no prediction).

**Figure 3:**
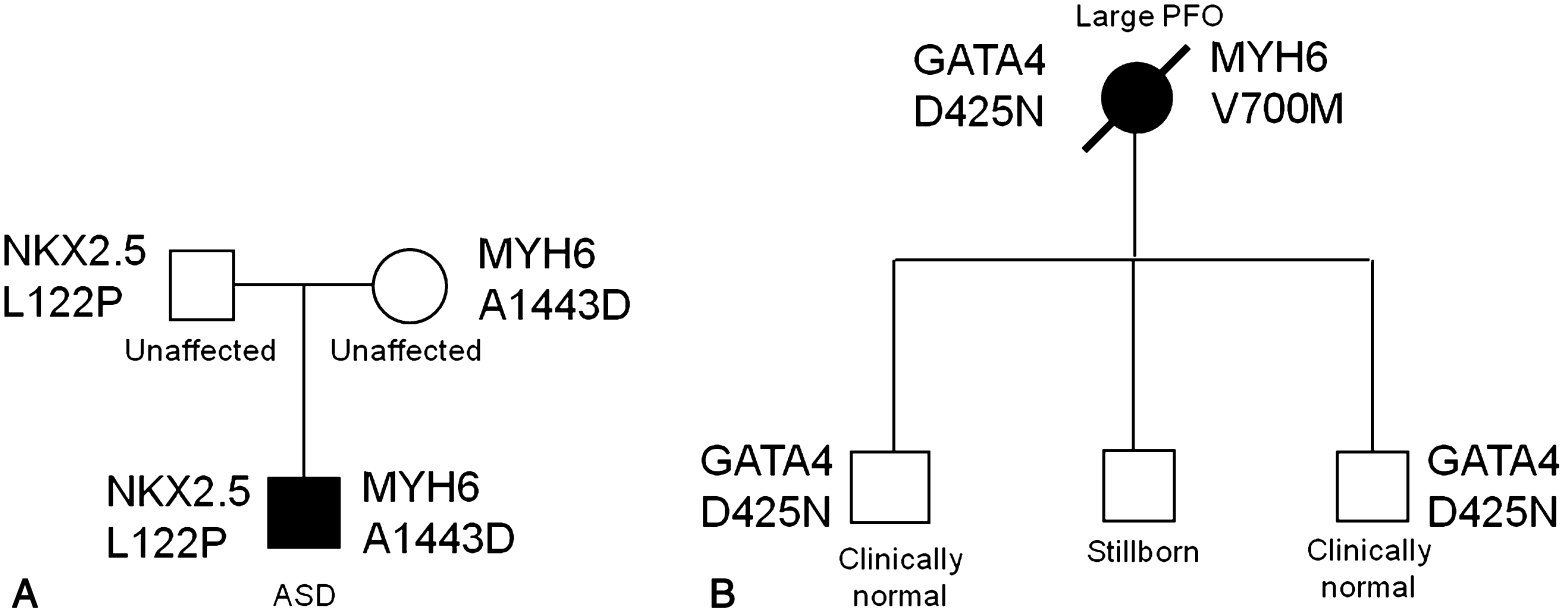
Pedigrees of the families CHD where multiple heterozygosity for the *NKX2.5*, *GATA4*, and *MYH6* genes was detected. ASD, atrial septal defect; PFO, patent foramen ovale.

The L122P change lies N-terminal to the homeodomain of NKX2-5. An analysis of the NKX2-5 wild-type and L122P mutant-derived protein sequences using the nnpredict program^36^ indicates that the variant occurs within a segment of the molecule that normally adopts an α-helical secondary structure that is disrupted in the mutant (Figure 2). This can be explained by the observation that, when a proline residue is located within an α-helical structure, the helix is kinked by approximately 30 degrees to prevent a steric clash between the pyrrolidine ring and the preceding carbonyl oxygens.^37^ This suggests that the private L122P mutation could affect the function of the NKX2-5 protein by modifying its three-dimensional conformation. This mutation was inherited from the father. In the same individual, there is another private mutation in a different gene, *MYH6*, (A1443D) which was transmitted by the mother (Figure 3A).^29^ Both parents are of white descent. The A1443D mutation introduces a negatively charged residue in a position where only noncharged amino acids exist in sarcomeric myosins from mammals, chick, zebrafish, *Xenopus*, and *Caenorhabditis elegans* and is predicted to interfere with the interaction between the two molecules of the myosin dimer or with other sarcomeric proteins.^29^ The frequency of the alleles encoding either L122P (*NKX2-5*) or A1443D (*MYH6*) in the CHD and control cohorts is 0.000619. The probability of both private alleles occurring in the same individual by chance, assuming the variants do not have causal relation to the phenotype is estimated to be 4.82 × 10^−7^. The most common phenotype resulting from mutations in either *NKX2-5* or *MYH6* is secundum ASD.^15^,^29^,^38^ As this patient has the same diagnosis, double heterozygocity could at least partially explain the development of the defect in the proband and the incomplete penetrance of the L122P and A1443D mutations in the parents. Incomplete penetrance is a common finding in documented cases of mutations causing Mendelian CHD.^4^ In the case of *NKX2-5*, recent data highlight the profound influence of modifier loci in the pathogenesis of single-gene cardiac malformation.^39^

**Figure 4:**
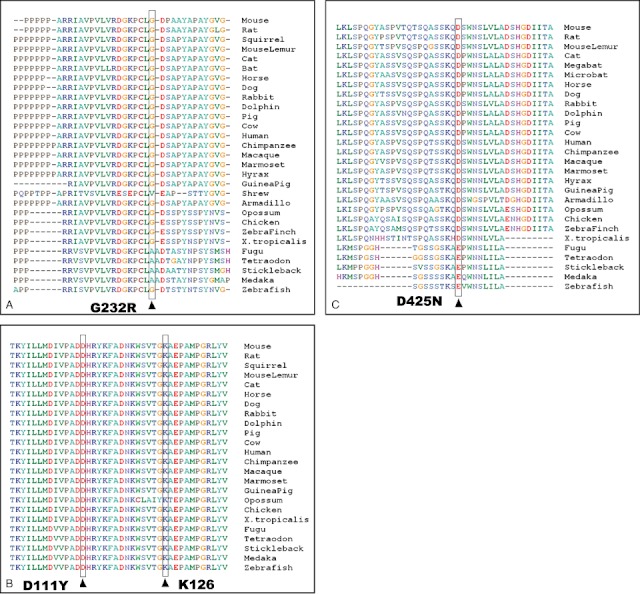
Multiple alignment of amino acid sequences of segments of the NKX2-5, GATA4, and TBX5 proteins from every vertebrate species available. (A) The *NKX2-5* G232R mutation introduces a positively charged arginine residue in a position where only noncharged amino acids are present in NKX2.5 vertebrate orthologs. (B) The *TBX5* D111Y mutation replaces a negatively charged aspartic acid residue with a noncharged tyrosine residue. Both amino acids forming a salt bridge predicted by the human TBX5 tridimensional structure (PDB: 2X6U) are universally conserved among TBX5 vertebrate orthologs. (C) The D425N *GATA4* mutation introduces a noncharged residue in a position where only negatively charged residues are present in every vertebrate GATA4 ortholog sequence available.

The novel NKX2-5 G232R mutation occurs in the C-terminal end of the NK2-specific domain (NK2-SD) and introduces a positively charged residue in a position where only noncharged residues exist in every vertebrate NKX2-5 ortholog sequence available (Figure 4A). The NK2-SD interaction with bone morphogenetic protein (BMP)-dependent mothers against decapentaplegic (SMAD) proteins is required for the binding of the homeodomain with the histone deacetylase (HDAC)/Sin3A complex.^40^ This domain is also thought to stabilize the interaction of the tinman domain to the corepressor Groucho proteins^41^ and to modulate, in an intramolecular fashion, the activity of the C-terminal activation domain.^42^ These data suggest that the G232R mutation could compromise the transcriptional-repressor activity of NKX2-5 and its responsiveness to BMP signaling.

**Figure 5:**
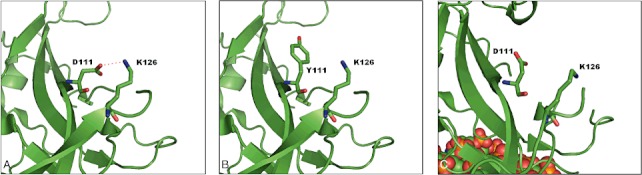
(A) Molecular model of human TBX5 protein free from nucleic acid based on PDB file 2X6U showing the salt bridge between the D111 and the K126 residues. (B) The D111Y change disrupts the salt bridge, as the negatively charged aspartic acid (D) residue is replaced by an uncharged tyrosine residue (Y). (C) Model of the T-box motif bound to nucleic acid based on PBD file 1H6F showing that during interaction with its target promoter, the side-chains of the D111 and K126 residues point to opposite directions, canceling the salt bridge.

The novel D111Y TBX5 variant occurs within the conserved T-box element of the molecule. A three-dimensional structure model of the human TBX5 protein (PDB: 2X6U) has been made available recently.^43^ This model allows more precise predictions of the consequences of the variant D111Y (Figure 5A). The structural model predicts that the salt bridge present between the K126 and D111 is disrupted when the aspartic acid residue (D) is replaced, as in the case of the D111Y variant, by an uncharged tyrosine (Y) residue (Figure 5B). A model of the human TBX3 bound to its target sequence (PDB: 1H6F)^44^ reveals that the side-chains of both K126 and D111 point to opposite directions during interaction with DNA, canceling the salt bridge (Figure 5C) that suggests that the D111Y variant could have an impact on the conformational change of TBX5 upon binding to its target promoters. Both residues involved in the salt bridge (D111 and K126) are universally conserved in every TBX5 vertebrate sequence available (Figure 4B). We suggest that these data warrant the inclusion of the TBX5 D111Y variant in future case-control studies for sporadic CHD, as this variant could confer susceptibility to the disease.

The D425N mutation of *GATA4* has been reported previously in three patients with secundum ASD, VSD, and tetralogy of Fallot.^34^,^35^ This variation replaces a negatively charged aspartic acid (D) residue for an uncharged asparagine (N) residue in a position where only negatively charged amino acids are found in GATA4 orthologs in wide range of vertebrate species (Figure 4C). The patient heterozygous for D425N also carries a private mutation in exon 18 of *MYH6*, a 2165G>A transition (V700M) (Figure 3B).^29^ This mutation is located within the segment of the *MYH6* gene encoding a part of the SH1 helix that is conserved in every myosin sequence available and is predicted to hinder the movements of the helix at different stages of the myosin cycle.^29^ Two clinically normal sons of the proposita are both heterozygous for the D425N *GATA4* mutation but do not carry the V700M *MYH6* mutation. The patient suffered a stillbirth but no sample from the product was available for analysis. The structure of this pedigree is consistent with complementation of both variants to contribute to the phenotype (Figure 3B).

## Conclusions

Mendelian nonsyndromic CHD is known to be caused by mutations of genes encoding transcription factors^4^ and, notably, muscle proteins expressed in the developing heart or great vessels like ACTC1,^45^ MYH6,^29^,^38^ MYH7,^46^ and MYH11.^47^,^48^ Here, we describe two novel mutations of *NKX2-5* associated with CHD. Also, we present cases where multiple heterozygosity of variants could contribute, by additive effects, to the development of individual cases of cardiac malformation. Our findings highlight the usefulness of multiple gene analysis in large patient CHD cohorts in order to identify variants whose combined effects may cause the phenotype.

Although array-based genome-wide association studies (GWAS) have been successful in identifying chromosomal segments and variants associated with complex phenotypes, in general, they only explain a small fraction of their heritability.^49^ Recent findings suggest that a great proportion of the “missing heritability” in GWAS studies can be masked by incomplete linkage disequilibrium between the causal variants and the single-nucleotide polymorphisms (SNPs) typed by the arrays, as causal variants would tend to have lower minor allele frequencies than the typed SNPs.^50^ Thus, we suggest there is a need for further detailed analysis of common, rare, and private variants of genes implicated in Mendelian CHD in association studies aimed at identifying the genetic causes of the more common, sporadic forms of the disease.
